# PCDHGA9 acts as a tumor suppressor to induce tumor cell apoptosis and autophagy and inhibit the EMT process in human gastric cancer

**DOI:** 10.1038/s41419-017-0189-y

**Published:** 2018-01-18

**Authors:** Junyong Weng, Jingbo Xiao, Yushuai Mi, Xu Fang, Yahuang Sun, Shanbao Li, Zhiwei Qin, Xu Li, Tingting Liu, Senlin Zhao, Lisheng Zhou, Yugang Wen

**Affiliations:** 1 Department of General Surgery, Shanghai General Hospital, School of Medicine, Shanghai Jiaotong University, 200080 Shanghai, China; 2 Shanghai Key Laboratory of Pancreatic Diseases & Department of Gastroenterology, Shanghai General Hospital, Shanghai Jiaotong University School of Medicine, 200080 Shanghai, China; 3 Department of General Surgery, The Second Hospital of Shandong University, Jinan, 250033 Shandong China; 4 Department of Pathology, Shanghai General Hospital, School of Medicine, Shanghai Jiaotong University, 200080 Shanghai, China

## Abstract

The results of a cDNA  array revealed that protocadherin gamma subfamily A, 9 (PCDHGA9) was significantly decreased in SGC-7901 gastric cancer (GC) cells compared with GES-1 normal gastric cells and was strongly associated with the Wnt/β-catenin and transforming growth factor-β (TGF-β)/Smad2/3 signaling pathway. As a member of the cadherin family, PCDHGA9 functions in both cell–cell adhesion and nuclear signaling. However, its role in tumorigenicity or metastasis has not been reported. In the present study, we found that PCDHGA9 was decreased in GC tissues compared with corresponding normal mucosae and its expression was correlated with the GC TNM stage, the UICC stage, differentiation, relapse, and metastasis (*p* < 0.01). Multivariate Cox analysis revealed that PCDHGA9 was an independent prognostic indicator for overall survival (OS) and disease-free survival (DFS) (*p* < 0.01). The effects of PCDHGA9 on GC tumor growth and metastasis were examined both in vivo and in vitro. PCDHGA9 knockdown promoted GC cell proliferation, migration, and invasion, whereas PCDHGA9 overexpression inhibited GC tumor growth and metastasis but induced apoptosis, autophagy, and G1 cell cycle arrest. Furthermore, PCDHGA9 suppressed epithelial–mesenchymal transition (EMT) induced by TGF-β, decreased the phosphorylation of Smad2/3, and inhibited the nuclear translocation of pSmad2/3. Our results suggest that PCDHGA9 might interact with β-catenin to prevent β-catenin from dissociating in the cytoplasm and translocating to the nucleus. Moreover, PCDHGA9 overexpression restrained cell proliferation and reduced the nuclear β-catenin, an indicator of Wnt/β-catenin pathway activation, suggesting that PCDHGA9 negatively regulates Wnt signaling. Together, these data indicate that PCDHGA9 acts as a tumor suppressor with anti-proliferative activity and anti-invasive ability, and the reduction of PCDHGA9 could serve as an independent prognostic biomarker in GC.

## Introduction

Gastric cancer (GC) is the third leading cause of cancer-related mortality worldwide and the fifth common cause of cancer globally^1^. Although extensive efforts to develop tactics for the management of GC patients have been made, the 5-year overall survival (OS) remains low, with a poor prognosis^2^. Thus, a deeper understanding of the mechanisms underlying GC development and metastasis may lead to innovative therapeutic strategies.

Analysis of the biological results from a complementary DNA (cDNA) microarray using Ingenuity Pathway Analysis (IPA) and the Database for Annotation, Visualization and Integration Discovery (DAVID) revealed protocadherin gamma subfamily A, 9 (PCDHGA9), belonged to protocadherins (Pcdhs), as a potential novel biomarker in GC. Pcdhs are the largest mammalian subgroup of the cadherin superfamily. However, little information is available about the association between Pcdhs and tumorigenesis or nuclear signaling. According to some models, β-catenin shows reduced binding to cadherins, mediating their impact in gene transcription. When bound, cadherins might function as competitors of nuclear signaling^3^. Therefore, the aim of the present study was to examine whether PCDHGA9 suppresses tumor cell proliferation via interaction with β-catenin that disrupt canonical Wnt signaling in GC.

Epithelial–mesenchymal transition (EMT) is a well-recognized biological process associated with tumor metastasis^4^. The underlying molecular mechanisms regulating EMT in tumor cells are complex and transforming growth factor-β (TGF-β) has been shown to induce EMT^5^. However, the mechanism of this regulation and induction in the context of PCDHGA9 in GC has not yet been investigated, although this mechanism may serve as a potential treatment target in GC.

In the present study, we examined PCDHGA9 expression in a tissue microarray (TMA) of samples from 83 patients to evaluate the relationship between its expression levels and the clinicopathological features in GC. We also used cell functional assays in vitro and in vivo to reveal the anti-carcinogenic mechanisms of PCDHGA9 in GC and explore its prognostic value. These findings provide the first evidence that PCDHGA9 acts as a tumor suppressor inducing tumor cell apoptosis, autophagy, and cell cycle arrest and reducing the EMT process in human GC.

## Results

### The results of high-throughput DNA microarray analysis via David and IPA

**Fig. 1:**
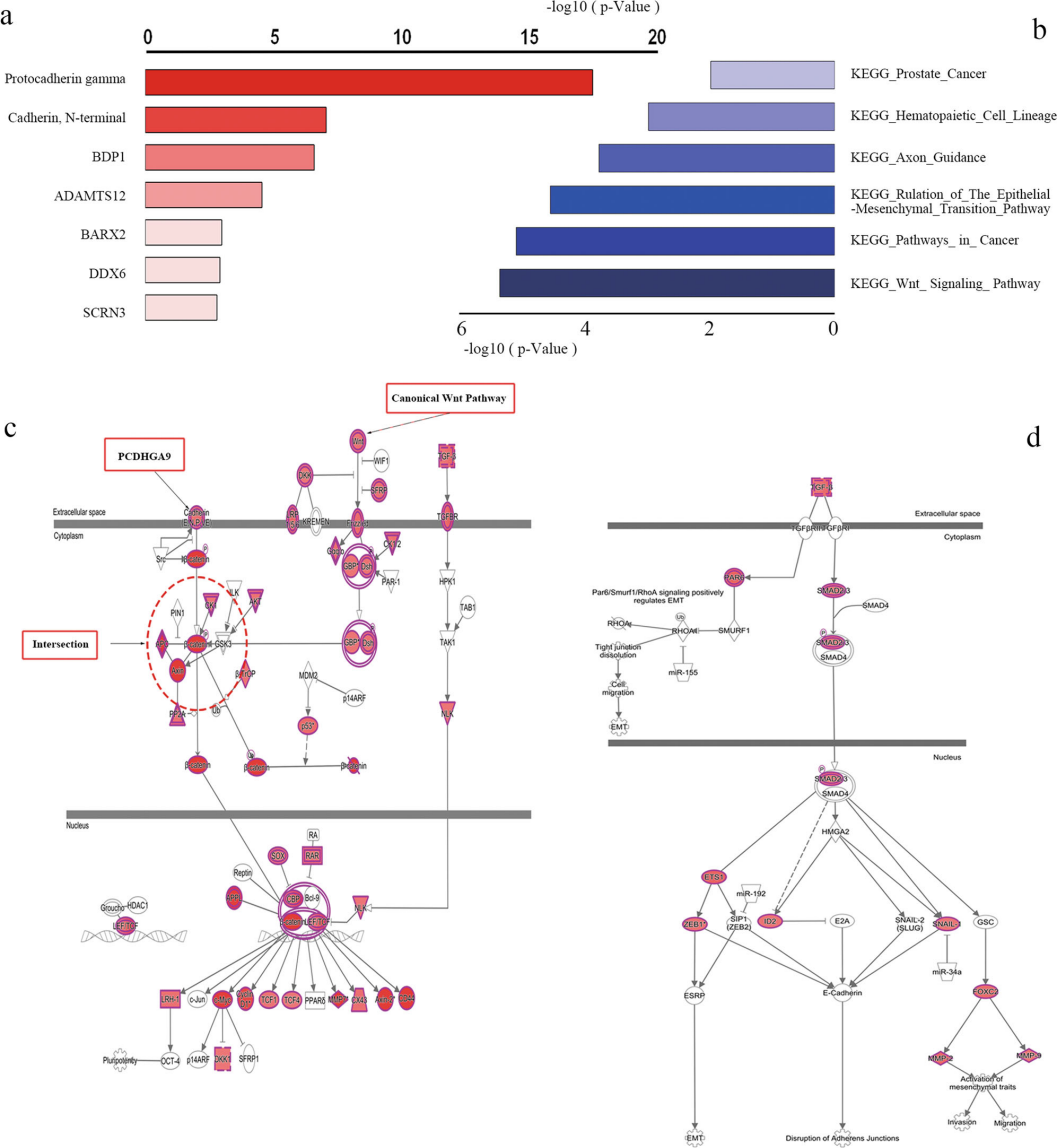
Analyses of the cDNA array via IPA and DAVID. **a** Protocadherin gamma received the top score in gene chip analysis using DAVID. **b** The Wnt signaling and EMT regulation pathways showed cluster enrichment based on DAVID and KEGG analysis. **c** IPA pathway enrichment showed that molecules of Wnt signaling were significantly altered and β-catenin was the shared factor between cadherin and Wnt signaling. **d** The TGF-β-induced EMT pathway was identified as a cluster via IPA. (Molecules with color indicate alterations detected using gene probes)

Microarray analysis using DAVID and IPA is commonly used for high-throughput bioinformatics. Using these two platforms, we examined the different gene expression patterns between normal cells and a high-invasion GC cell line. PCDHGA9 was the most valuable gene or protein related to cell–cell identification and adhesion (Fig. 1a). Moreover, the Wnt/β-catenin and TGF-β-induced EMT signaling pathway showed considerable *p*-values (Figs. 1b–d), and the canonical molecules associated with these two pathways were significantly modulated, consistent with PCDHGA9 expression.

### PCDHGA9 expression is reduced in human GC tissue

**Fig. 2:**
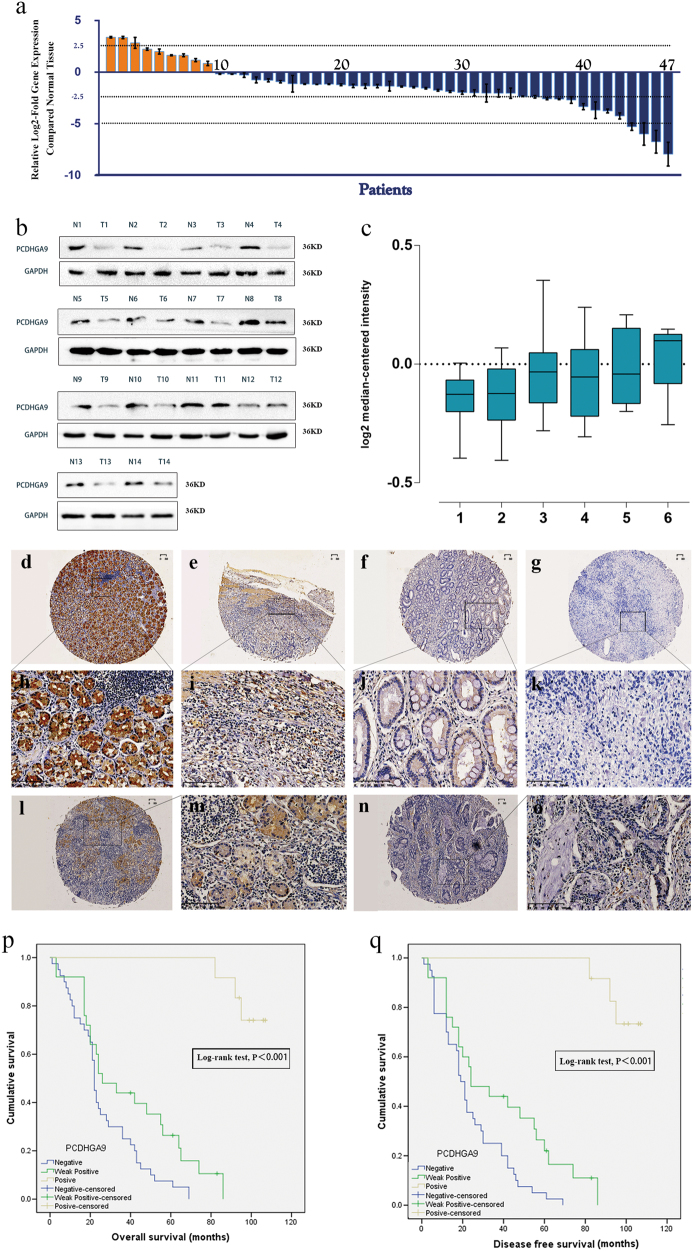
The expression of PCDHGA9 in GC tissues and corresponding normal mucosae and Kaplan–Meier analysis with a log-rank test of survival. **a** Quantitative real-time PCR showed that PCDHGA9 expression was decreased in 47 human GC tissue samples compared with paired normal mucosae. **b** Western blot analysis was used to detect PCDHGA9 protein expression in 14 representative paired GC tissue specimens. **c** A logarithmic 2^-∆∆T^ scale was used to represent the fold-change in PCDHGA9 mRNA expression in the microarray data set from the Oncomine database: Cho gastric, grouped by (1) no value, (2) gastric intestinal type adenocarcinoma, (3) gastric mixed adenocarcinoma, (4) diffuse gastric adenocarcinoma, (5) gastric adenocarcinoma, (6) gastrointestinal stromal tumor. **d**, **h** PCDHGA9 protein expression was significantly higher in normal mucosa compared with GC tissue, with strong PCDHGA9 staining in the normal gastric epithelium. **e**, **i** Moderate PCDHGA9 staining was observed in well-differentiated GC tissue. **f**, **j** Weak PCDHGA9 staining was observed in moderately differentiated GC. **g**, **k** Negative PCDHGA9 staining was observed in poorly differentiated GC. **l**, **m** Stronger PCDHGA9 staining was observed in GC tissues with no- metastasis. **n**, **o** Compared with M0-stage GC tissues, PCDHGA9 was expressed at very low levels in GC tissue with metastasis. **p** OS and **q** DFS were significantly shorter in patients with PCDHGA9-negative tumors than in those with PCDHGA9-positive tumors (*p* < 0.001 for both, log-rank test). *p*-Values were calculated using log-rank test, and *p* < 0.05 was considered significant. **d**, **e**, **f**, **g**, **l**, **n** Original magnification: ×40; **h**, **i**, **j**, **k**, **m**, **o** Original magnification: ×200

The results of real-time PCR indicated that the messenger RNA (mRNA) level of PCDHGA9 was significantly decreased in 38 (80.85%) GC samples compared with that in the nonmalignant samples (Fig. 2a). Western blot analysis further confirmed that PCDHGA9 protein was decreased in GC tissues, consistent with the microarray data sets from the Oncomine database (Figs. 2b, c).

### PCDHGA9 expression is correlated with GC clinicopathological characteristics

Immunohistochemical (IHC) analysis of a TMA containing 83 cases of primary GC tissue with corresponding normal mucosa was used to explore the relationship between PCDHGA9 expression and the clinical characteristics of GC patients, as summarized in Table 1. PCDHGA9 was mainly expressed in normal mucosa, and the staining was significantly stronger than that in tumor tissue (Table 2). According to the results of the staining experiments, PCDHGA9 expression could be divided into three categories, positive 44/83 (53.0%) in normal mucosae (Figs. 2d, h) versus 13/83 (15.7%) in tumor tissues (Figs. 2e, i); weak positive 22/83 (26.5%) in paracancerous mucosae versus 29/83 (34.9%) in GC specimens (Figs. 2f, j); and negative 17/83 (20.5%) versus 41/83 (49.4%) in tumor samples (Figs. 2g, k). Moreover, the staining level in patients with metastasis (Figs. 2n, o) was weaker than that in patients without metastasis (Figs. 2l, m). These data verified that PCDHGA9 expression was decreased in GC tissues compared with adjacent mucosa (*p* < 0.001). The reduction of PCDHGA9 expression in GC was remarkably correlated with the T stage (*p* < 0.001), N stage (*p *= 0.003), M stage (*p *= 0.006), UICC stage (*p* < 0.001), histological differentiation (*p* = 0.001), and tumor relapse (*p* = 0.001, Table 1). These results indicated that PCDHGA9 expression was associated with an aggressive GC phenotype.

**Table 1:** Association between PCDHGA9 and clinicopathological features in gastric cancer (*n* = 83)

	*N*	PCDHGA9 expression			*P*-value
		Negative (41)	Weak positive (29)	Strong positive (13)	
*Age (years)*					0.985
<65	39	19 (48.7)	14 (35.9)	6 (15.4)	
≥65	44	22 (50.0)	15 (34.1)	7 (15.9)	
*Gender*					0.587
Male	55	28 (50.9)	20 (36.4)	7 (12.7)	
Female	28	13 (46.4)	9 (32.1)	6 (21.4)	
*Tumor location*					0.351
Gastric fundus	9	3 (33.3)	3 (33.3)	3 (33.3)	
Gastric corpus	73	38 (52.1)	25 (34.3)	10 (13.7)	
Pylorus	1	0 (0)	1 (100.0)	0 (0)	
*Tumor sizes (cm)*					0.556
<3	10	4 (40.0)	5 (50.0)	1 (10.0)	
≥3	73	37 (50.7)	24 (32.9)	12 (16.4)	
*T stage*					<0.001
T1	8	0 (0)	2 (25.0)	6 (75.0)	
T2	8	3 (37.5)	0 (0)	5 (62.5)	
T3	53	28 (52.8)	23 (43.4)	2 (3.8)	
T4	14	10 (71.4)	4 (28.6)	0 (0)	
*N stage*					0.003
N0	19	3 (15.8)	11 (57.9)	5 (26.3)	
N1	14	5 (35.7)	5 (35.7)	4 (28.6)	
N2	24	16 (66.7)	4 (16.7)	4 (16.7)	
N3	26	17 (65.4)	9 (34.6)	0 (0)	
*M stage*					0.006
M0	74	32 (43.2)	29 (39.2)	13 (17.6)	
M1	9	9 (100.0)	0 (0)	0 (0)	
*UICC stage*					<0.001
I	8	0 (0)	1 (12.5)	7 (87.5)	
II	22	4 (18.2)	13 (59.1)	5 (22.7)	
III	43	28 (65.1)	14 (32.6)	1 (2.3)	
IV	10	9 (90.0)	1 (10.0)	0 (0)	
*Vessel invasion*					0.290
No	70	32 (45.7)	26 (37.1)	12 (17.1)	
Yes	13	9 (69.2)	3 (23.1)	1 (7.7)	
*Nerve invasion*					0.461
No	80	40 (50.0)	27 (33.8)	13 (1.25)	
Yes	3	1 (33.3)	2 (66.7)	0 (0)	
*Differentiation*					0.001
High	1	0 (0)	0 (0)	1 (100)	
Moderate	36	13 (36.1)	16 (44.4)	7 (19.4)	
Low	46	28 (60.9)	13 (28.3)	5 (10.9)	
*Relapse*					0.001
No	44	15 (34.1)	17 (38.6)	12 (27.3)	
Yes	39	26 (66.7)	12 (30.8)	1 (2.6)

**Table 2:** Expression of PCDHGA9 in gastric normal mucosa and cancerous tissue

Tissue sample	*N*	Expression of PCDHGA9			*P*-value
		Negative(%)	Weak positive(%)	Positive(%)	
Normal mucosa	83	17 (20.5)	22 (26.5)	44 (53.0)	<0.001
GC tissue	83	41 (49.4)	29 (34.9)	13 (15.7)	

*GC* gastric cancer

### Decreased PCDHGA9 expression predicts poor clinical outcome in GC

The correlation between PCDGA9 expression and OS or disease-free survival (DFS) was assessed using Kaplan–Meier survival analysis. PCDHGA9-negative patients showed poorer OS (*p* < 0.001, Fig. 2p) and DFS (*p* < 0.001, Fig. 2q) than those with higher PCDHGA9 expression. Subsequently, multivariate analysis demonstrated that PCDHGA9 expression remained an independent prognostic factor for poor outcomes in patients with GC (Table 3). In summary, these data identify negative PCDHGA9 expression as an independent prognostic biomarker for poor prognosis in GC patients.

**Table 3:** Univariate and multivariate Cox proportional hazard models for overall survival after surgery

	**Overall survival**			
	Univariate analysis		Multivariate analysis	
	HR (95% CI)	*P*-value	HR (95% CI)	*P*-value
*Age (years)*				
<65	–			
≥65	0.830 (0.509–1.355)	0.457		
*Gender*				
Male	–			
Female	0.753 (0.444–1.277)	0.292		
*Tumor location*				
Gastric fundus	–			
Gastric corpus	1.089 (0.496–2.393)	0.832		
Pylorus	4.034 (0.478–34.077)	0.200		
*Tumor sizes (cm)*				
<3	–			
≥3	1.285 (0.467–3.537)	0.627		
*T stage*				
T1	–			
T2	0.863 (0.215–3.460)	0.835		
T3	3.457 (1.209–9.883)	0.021		
T4	3.770 (1.190–11.942)	0.024		
*N stage*				
N0	–			
N1	1.540 (0.764–3.100)	0.227		
N2	2.616 (1.302–5.255)	0.007		
N3	0.000 (0.000–0.000)	0.969		
*M stage*				
M0	–			
M1	2.562 (1.218–5.391)	0.013		
*UICC stage*				
I	–			
II	4.521 (1.032–19.798)	0.045		
III	9.787 (2.298–41.671)	0.002		
IV	13.481 (2.833–64.142)	0.001		
*Vessel invasion*				
No	–			
Yes	1.194 (0.649–2.196)	0.568		
*Nerve invasion*				
No	–			
Yes	0.861 (0.269–2.759)	0.802		
*Differentiation*				
High	–			
Moderate	1.488 (0.201–11.013)	0.697		
Low	1.838 (0.251–13.478)	0.550		
*Relapse*				
No	–			
Yes	2.550 (1.521–4.276)	<0.001		
*PCDHGA9*				
Negative	–		–	
Weak	0.522 (0.301–0.905)	0.021	0.461 (0.225–0.946)	0.035
Positive	0.013 (0.002–0.104)	<0.001	0.003 (0.000–0.050)	<0.001

*HR* hazard ratio, *CI* confidence interval

### Overexpression of PCDHGA9 significantly suppresses GC cell migration and invasion

**Fig. 3:**
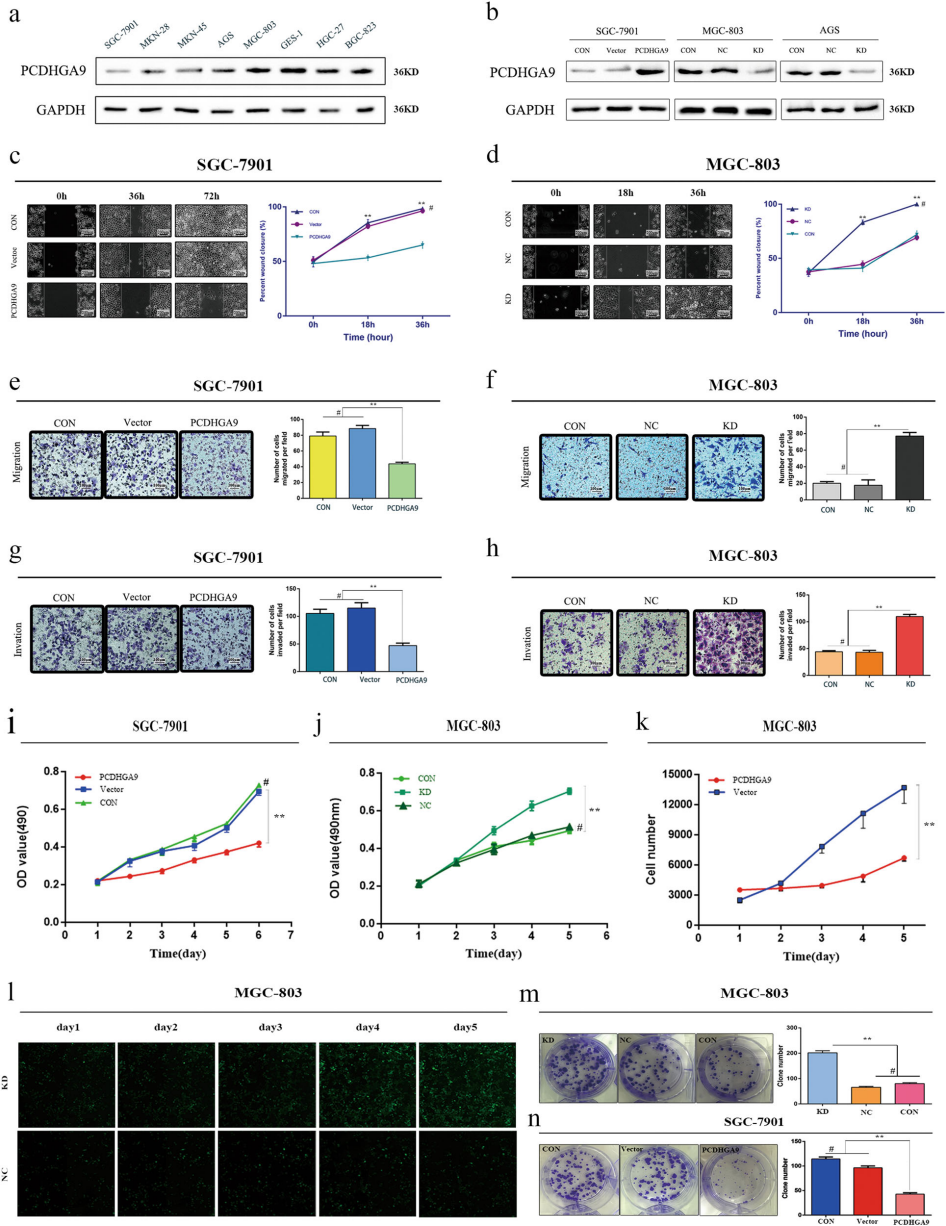
PCDHGA9 expression in cell lines and functional assays in vitro. **a** PCDHGA9 protein level in a gastric mucosa cell line (GES-1) and 7 GC cell lines. **b** SGC-7901, MGC-803, and AGS cells transfected with PCDHGA9 overexpression or downregulation vectors were validated using western blotting. GAPDH was used to normalize protein expression. Overexpression or knockdown of PCDHGA9 suppressed or elevated GC cell proliferation, migration and invasion, respectively. **c**, **d** Wound healing. **e**, **f** Migration ability. **g**, **h** Invasion ability. **i**, **j** CCK8 assays. **k**, **l** The Celigo image cytometer was used to count the cell number, showing that knockdown of PCDHGA9 promoted cell proliferation. **m**, **n** Colony formation assay. (**p < 0.01). Original magnification: ×100. CON, control group without any infection; Vector group transfected with empty vector; NC, infected with negative lentivirus; KD, infected with Lenti-shRNA (^#^p > 0.05)

To investigate the influence of PCDHGA9 expression on the biological behavior of GC cells, we selected SGC-7901 cells to generate an overexpression cell model (Fig. 3b). Wound-healing assays and transwell assays showed that overexpression of PCDHGA9 could significantly inhibit the migration and invasion of SGC-7901 cells (Figs. 3c, e, g). On the contrary, PCDHGA9 knockdown evidently enhanced the wound healing, migration and invasion of MGC-803 cells (Figs. 3d, f, h) and AGS cells (Supplementary Figure 1a, b, c).

### Different expression patterns of PCDHGA9 differently affect the EMT process of GC cells

**Fig. 4:**
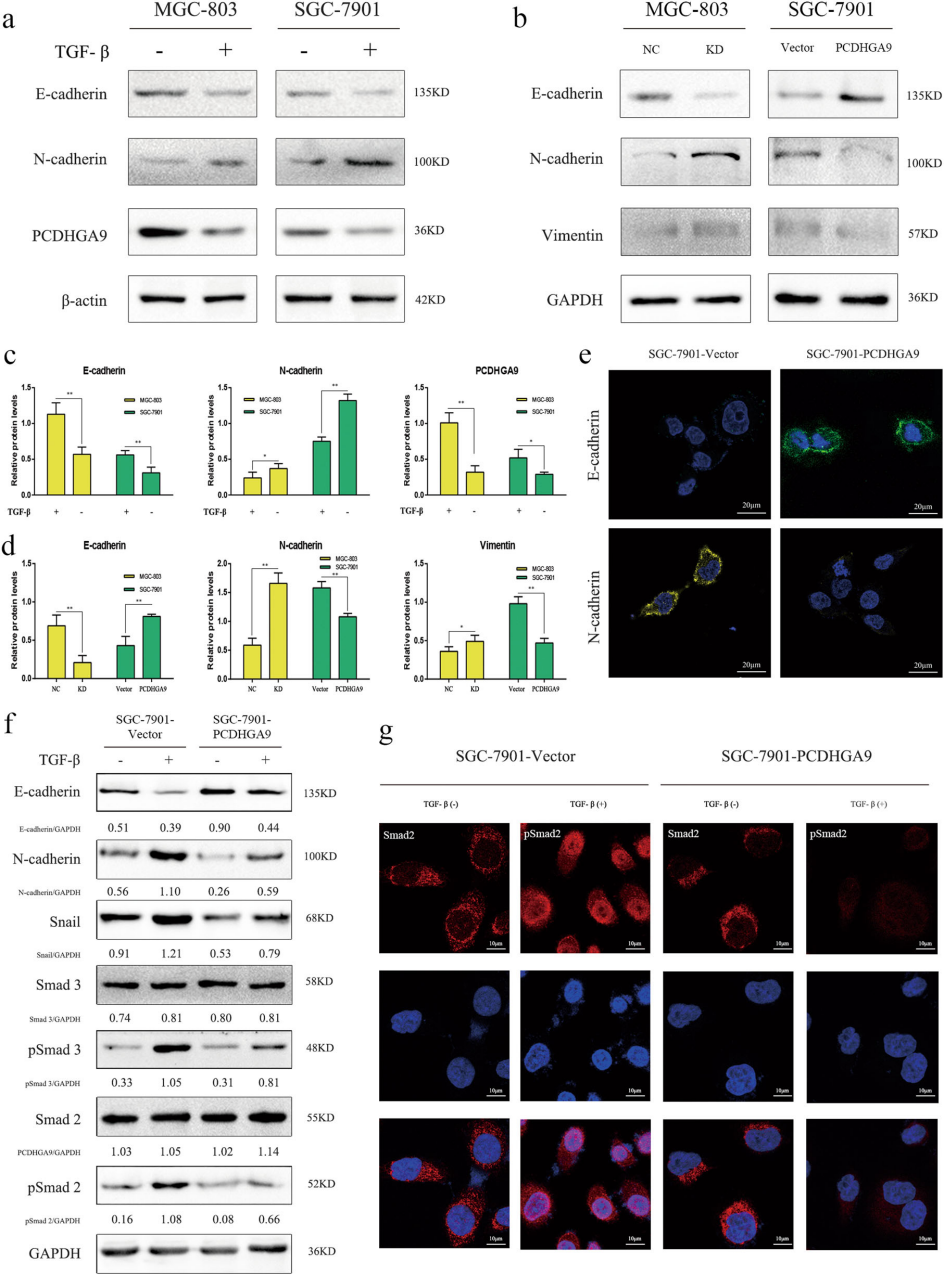
Overexpression of PCDHGA9 inhibited the EMT process induced by TGF-β. **a**, **c** PCDHGA9 was downregulated in artificial EMT models induced by TGF-β in GC cells. Up or downregulated expression of E-cadherin and N-cadherin was verified in the EMT models. β-Actin was used to normalize protein expression. **b**, **d** Western blot analysis of EMT markers (E-cadherin, N-cadherin, and vimentin) in the PCDHGA9 knockdown or overexpression cells compared with the control group. GAPDH was used to normalize protein expression. **e** Immunofluorescence showed that E-cadherin and N-cadherin expression levels were strengthened or weakened, respectively, in PCDHGA9-overexpressing SGC-7901 cells via laser confocal microscopy. **f** Western blot suggested that TGF-β induced EMT, upregulated N-cadherin, Snail and pSmad2/3, downregulated E-cadherin and did not alter Smad2/3 expression significantly, whereas overexpression of PCDHGA9 weakened this tendency and inhibited EMT in GC cells. GAPDH was used to normalize protein expression. **g** Immunofluorescence analysis via laser confocal microscopy indicated that overexpression of PCDHGA9 could decrease the phosphorylation of Smad2 and suppress the nuclear translocation of pSmad2 (^*^p < 0.05 and ^**^p < 0.01)

TGF-β1 notably induced EMT in GC cells after 2 h, significantly increasing N-cadherin and decreasing E-cadherin expression. Interestingly, PCDHGA9 expression was simultaneously reduced (Fig. 4a). We hypothesized that PCDHGA9 inhibited EMT, and we further investigated whether altering the expression of PCDHGA9 could impact the phenotypes of GC cell lines. PCDHGA9 expression augmented the levels of E-cadherin and reduced the levels of vimentin and N-cadherin in SGC-7901 cells (Fig. 4b, right panel). These results indicated that PCDHGA9 inhibited the EMT process. Confocal immunofluorescence analysis further confirmed this hypothesis (Fig. 4e). In addition, western blot analysis revealed that PCDHGA9 knockdown in MGC-803 and AGS cells generated the opposite results (Fig. 4b, left panel, Supplementary Figure 3).

### PCDHGA9 suppresses GC cell EMT and metastasis through TGF-β/Smad2/3 signaling

PCDHGA9 significantly suppressed EMT in GC cells. According to pathway enrichment analysis via IPA and DAVID, we hypothesized that PCDHGA9 exerted its effects on EMT through TGF-β/Smad2/3 signaling (Fig. 1d). Enhanced N-cadherin or Snail and decreased E-cadherin expression in response to TGF-β1 were observed in both PCDHGA9 -overexpressing or vector SGC-7901 cells, indicating that TGF-β evidently induced EMT. Moreover, Smad2/3 retained a consistent level of expression, and pSmad2/3 was detected after the addition of TGF-β1. Interestingly, PCDHGA9 overexpression reduced Smad2/3 phosphorylation to some extent and inhibited pSmad2/3 nuclear translocation via western blot analysis (Fig. 4f) and laser confocal microscopy (Fig. 4g). Collectively, these findings indicate that PCDHGA9 plays a role in TGF-β/Smad2/3 signaling and suppresses EMT in GC cells.

### PCDHGA9 regulates cell proliferation and growth in GC

Compared with control cells, the ectopic overexpression of PCDHGA9 significantly inhibited the proliferation of GC cells in a time-dependent manner (Fig. 3i) and distinctly suppressed colony formation in GC cells (Fig. 3n). In addition, the loss of PCDHGA9 accelerated cell proliferation and growth in MGC-803 and AGS cells (Figs. 3j-m, Supplementary Figure 2). These results suggest that PCDHGA9 acts as a tumor suppressor in GC partly through regulating cell proliferation and growth.

### Overexpression of PCDHGA9 promotes the apoptosis of GC cells and induces G1 cell cycle arrest

**Fig. 5:**
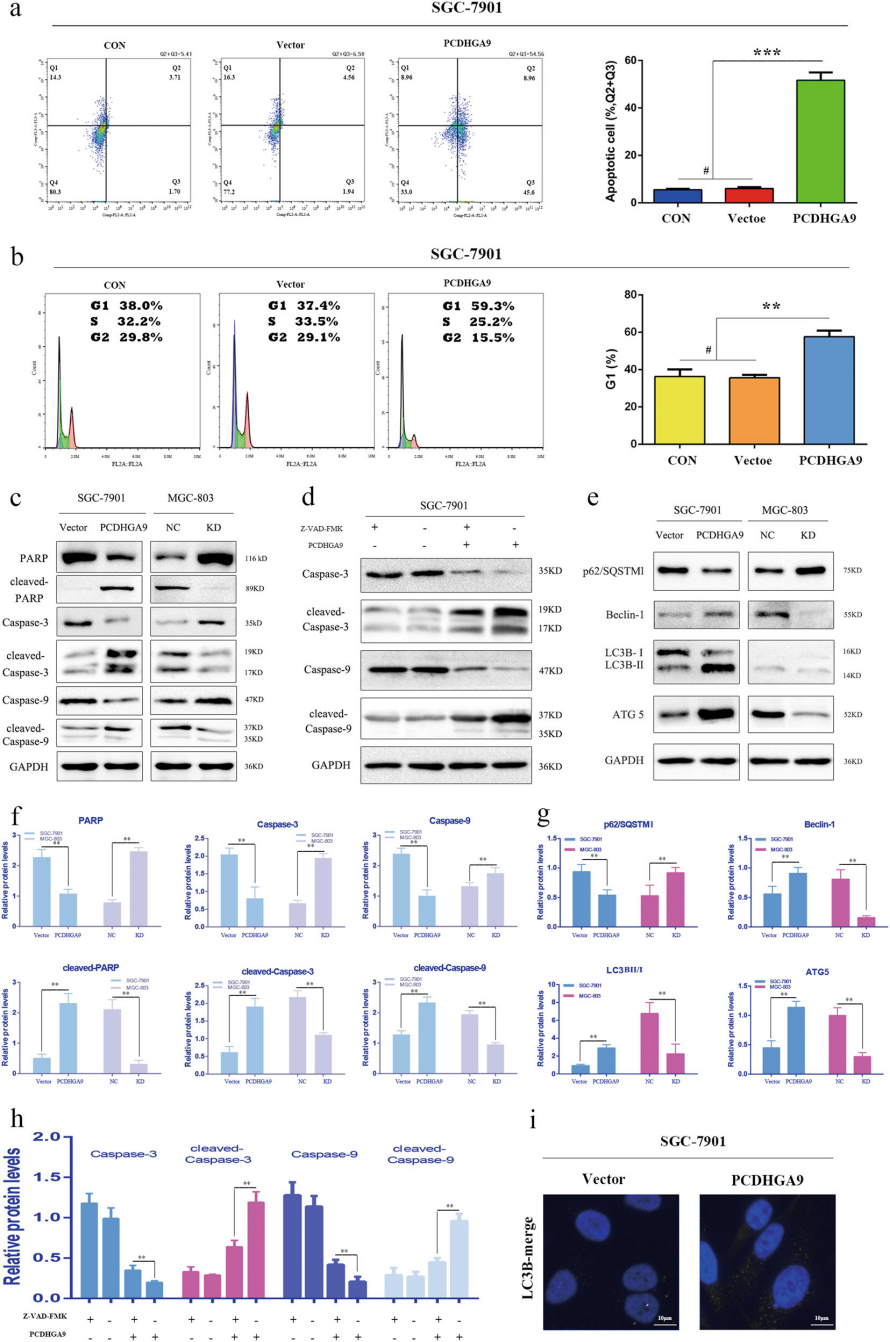
Overexpression PCDHGA9 induced GC cell apoptosis, cell cycle arrest, and autophagy. **a** Representative images of apoptosis analysis of SGC-7901 cells (CON, Vector, Overexpression) showed that overexpression of PCDHGA9 induced GC cell apoptosis. **b** Representative images of cell cycle analysis. PCDHGA9 induced G1 phase arrest. **c**, **f** Western blot analysis confirmed that PCDHGA9 induced altered expression of apoptosis-related proteins. **d**, **h** Western blot analysis determined that 10 μg/ml V-ZAD-FMK inhibits the activation of caspases. **e**, **g** Autophagy-related proteins were evaluated by western blot analysis in SGC-7901 and MGC-803 cells. GAPDH was used to normalize protein expression. **i** The level and cellular localization of LC3B was determined in vector-alone and PCDHGA9-overexpressing cells. (**p < 0.01, ***p < 0.001, ^#^p > 0.05)

**Fig. 6:**
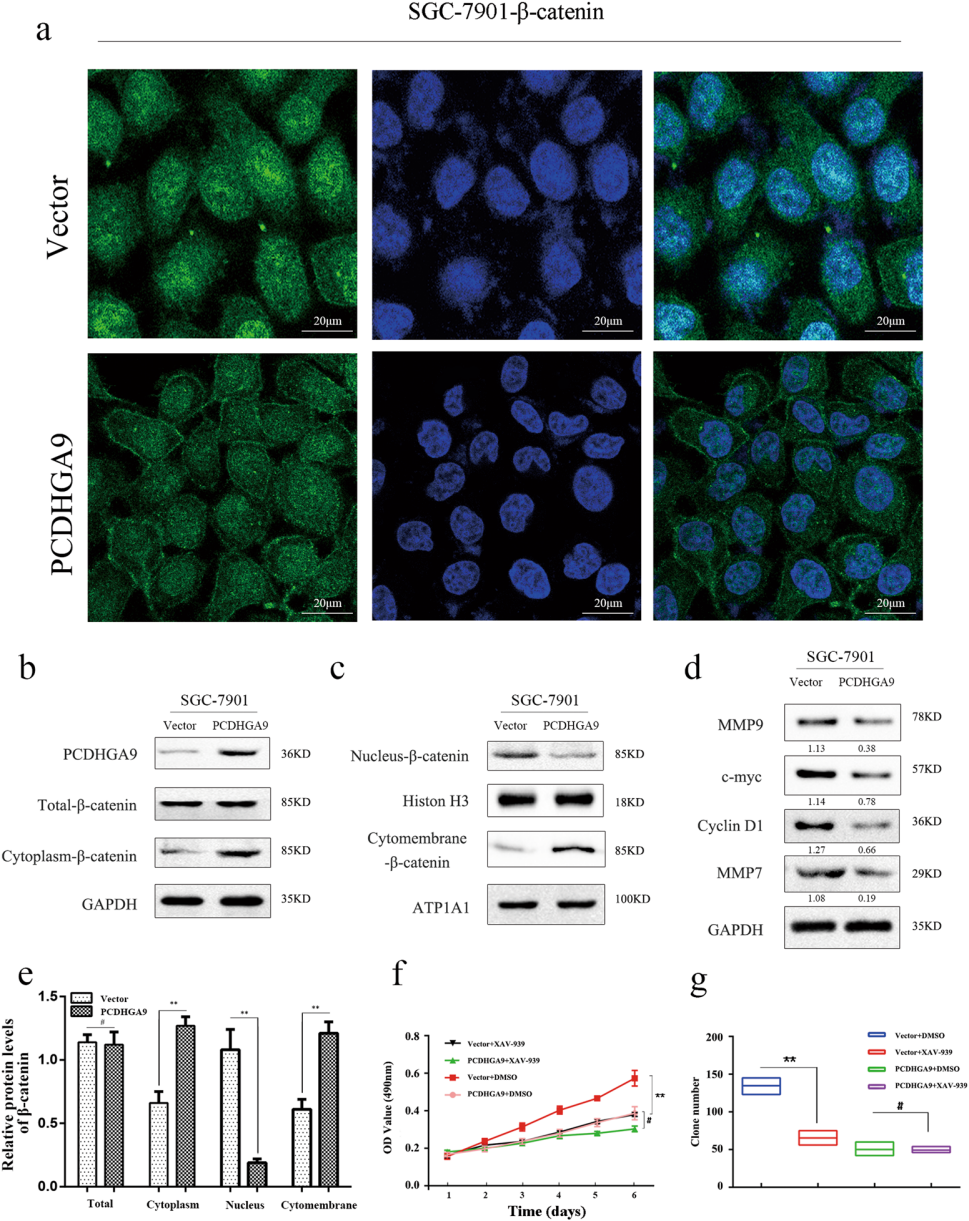
PCDHGA9 inhibited the proliferation of GC cells by interacting with β-catenin and negative regulating the Wnt signaling pathway. **a**, **b**, **c**, **e** The cellular locations of β-catenin were detected by immunofluorescence and western blot. GAPDH, Histone H3, and ATP1A1 were used to normalize protein expression in total lysates and nuclear and membrane fractions, respectively. SGC-7901 cells with PCDHGA9 overexpression or vector control cells were grown in media with or without 10 μg/ml inhibitor of β-catenin (XAV-939). **d** Western blot analysis of cell cycle-related proteins (c-myc and cyclin D1) and biomarkers of cell invasion (MMP9 and MMP7) was shown, and that c-myc, cyclin D1, and MMP7 were downstream target molecules of the Wnt/β-catenin pathway. **f** CCK8 assays and **g** colony formation assays were used to examine the GC cells’ proliferation ability. **a** Original magnification ×400 (**p < 0.01, ^#^*p* > 0.05)

To identify the mechanism for the anti-proliferative effects of PCDHGA9 in GC cells, the apoptosis and cell cycle status of overexpression and control SGC-7901 cells were analyzed. The results of flow cytometry indicated that PCDHGA9 remarkably induced cell apoptosis and increased the percentage of apoptotic SGC-7901 cells (Fig. 5a), consistent with level of apoptotic-related proteins by western blot analysis (Figs. 5c, f). The activation of caspase-3 and caspase-9 caused by PCDHGA9 were significantly suppressed using the Z-VAD-FMK, a broad-range caspase inhibitor (Figs. 5d, h). Next, we determined whether tumor cell growth inhibition by PCDHGA9 was related to cell cycle arrest. The flow cytometry results showed that PCDHGA9 overexpression induced G1 phase cell cycle arrest in SGC-7901 cells (Fig. 5b) consistent with the result that PCDHGA9 reduced the expression of c-myc and cyclin D1 (Fig. 6d).

### Upregulation of PCDHGA9 induces the autophagy of GC cells

Autophagic cell death is different from apoptosis, although both mechanisms have been implicated in tumorigenesis and development. To determine whether overexpression of PCDHGA9 could induce autophagy, we evaluated the conversion of LC3B, SQSTM1/p62, Beclin-1, and ATG5 using western blotting. Then, the results indicate that PCDHGA9 suppresses GC cell proliferation, at least in part, through the induction of autophagy. Moreover, the obvious dot-like fluorescence of LC3B was observed in PCDHGA9- transfected cells (Fig. 5i). Although the precise mechanism for PCDHGA9-regulated autophagy in GC cells remains unknown, these results suggest that PCDHGA9 restrains tumor proliferation through the induction of autophagy in GC cells.

### PCDHGA9 suppresses the nuclear translocation of β-catenin to decrease the proliferation of GC cells but not via the canonical Wnt/β-catenin pathway

β-Catenin is typically integrated with cadherins as a part of the protein complex at so-called adherens junctions, whereas dissociated β-catenin is targeted for ubiquitination and degradation by the destruction complex. However, the activation of Wnt signaling can stabilize β-catenin for eventual translocation to the nucleus and subsequent Lymphoid enhancer-binding factor (LEF)/Transcription factor (TCF) binding to activate several genes, as shown in Fig. 1c. Our immunofluorescence analysis showed that compared with the control group, β-catenin membrane expression was augmented and its nuclear translocation was reduced in PCDHGA9- overexpressing cells (Fig. 6a). We hypothesized that PCDHGA9, similar to E-cadherin, interacts with β**-**catenin and decreases its nuclear translocation to antagonize Wnt signaling. To examine this hypothesis, we extracted total cell lysates, cytomembrane, and nuclear proteins, respectively, and then measured the β-catenin levels using western blot analysis. We observed no association between total cellular β-catenin and PCDHGA9 expression. Nevertheless, control cells showed increased nuclear β-catenin, an indicator of Wnt/β-catenin pathway activation, and decreased cytomembrane or cytoplasmic β-catenin compared with PCDHGA9-overexpressing cells (Figs. 6b, c), supporting the role of PCDHGA9 as a negative regulator of canonical Wnt signaling, consistent with the aforementioned hypothesis. Moreover, cyclin D1, c-myc, and MMP7, as downstream effectors of canonical Wnt signaling, showed a significant negative correlation with PCDHGA9 expression (Fig. 6d), suggesting that PCDHGA9 may suppress GC cell proliferation via suppression of the canonical Wnt pathway.

Using the β**-**catenin decomposed reagent XAV-939, the proliferation and colony formation of PCDHGA9-overexpressing cells was not remarkably suppressed, whereas control cells were markedly inhibited (*p* < 0.01, Figs. 6f, g). Taken together, these data indicate that PCDHGA9 suppression of GC progression involves interactions with β**-**catenin and that to some extent compete with canonical Wnt signaling.

### PCDHGA9 inhibits tumor growth and metastasis in nude mice

**Fig. 7:**
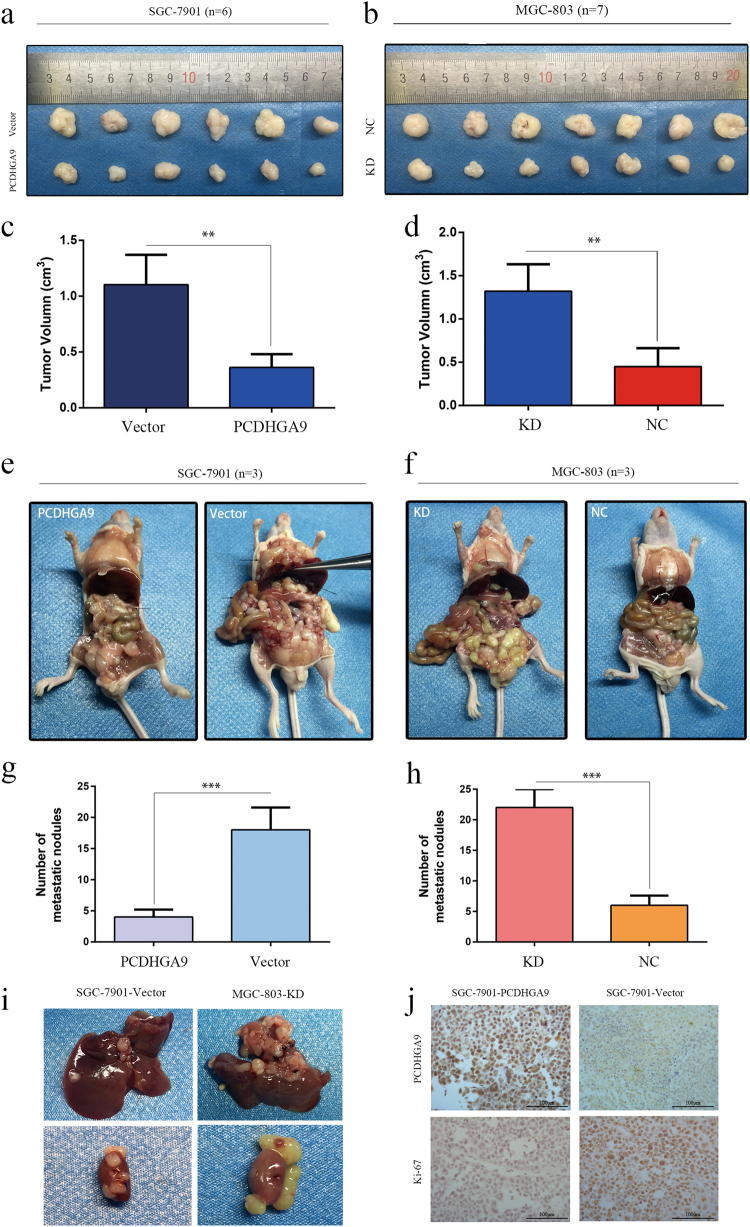
Overexpression or knockdown of PCDHGA9 inhibited or promoted tumor formation and metastasis of GC cells in nude mice, respectively. **a**–**h** Representative photographs of subcutaneous tumor xenografts and peritoneal dissemination in nude mice. The arrowheads point to tumor nodules or liver metastasis nodes. The subcutaneous tumors in the control group were remarkably larger than those in the overexpression group. The number of peritoneal dissemination metastatic nodules in the control group was significantly higher than that in the PCDHGA9 overexpression group. **i** Representative images showed liver and kidney metastasis with SGC-7901-Vector and MGC-803-KD cell peritoneal xenografts. **j** IHC staining analyses show the staining of PCDHGA9 and Ki-67 in subcutaneous tumors. Overexpression of PCDHGA9 significantly weakened Ki-67 staining. Original magnification ×200. (**p < 0.01, ***p < 0.001)

To verify the effect of PCDHGA9 on the tumorigenicity of GC cells, a xenograft tumor growth assay was performed. Control and stable PCDHGA9 overexpression cell lines obtained, and the SGC-7901-vector and SGC-7901-PCDHGA9 cells were subcutaneously inoculated into nude BALB/c mice. The formation of tumors was monitored after injection. At 24 days after implantation, we observed that the sizes of SGC-7901-PCDHGA9 cell-derived xenograft tumors were significantly smaller than those of SGC-7901-vector cell-derived tumors (Figs. 7a, c). IHC analysis showed that the expression of PCDHGA9 was increased, whereas that of Ki-67 was decreased, in the PCDHGA9 overexpression group (Fig. 7j). These results show that PCDHGA9 overexpression inhibited the tumorigenicity of GC cells.

The role of the PCDHGA9 metastatic phenotype was examined in vivo by implanting PCDHGA9-overexpressing cells and control cells into the peritoneal cavity of nude mice. We observed that mice bearing the SGC-7901-vector cells were much weaker and had larger abdomens filled with ascites. Necropsy revealed that the control cells extensively colonized the visceral organs and formed multiple metastatic nodules (Fig. 7e), whereas the number of metastatic nodules was reduced in mice bearing PCDHGA9-overexpressing cells. Furthermore, organs metastases were observed in the control group, revealing that overexpression of PCDHGA9 inhibited tumor colonization, migration and invasion (Fig. 7i). For further confirmation, we repeated these experiments using PCDHGA9-KD-MGC-803 cells and observed that the knockdown PCDHGA9 promoted proliferation and metastasis in GC (Figs. 7b, d, f, h, i). These results were consistent with the assays performed in vitro.

## Discussion

GC is one of the most common causes of cancer mortality worldwide, and metastasis and recurrence are the main process leading to the poor prognosis of GC patients^1^. Although several genes have been functionally characterized in recent studies, outstanding biomarkers for the prediction of GC outcomes are still lacking^6^. Here, we used cDNA microarrays and DAVID and IPA analyses to identify the novel biomarker PCDHGA9 in GC. We further explored the significance of PCDHGA9 in GC, which showed remarkably decreased expression in GC with a minimum *p*-value and comprehensive enrichment by IPA and DAVID. To examine the putative function of PCDHGA9, we investigated the relationship between PCDHGA9 and the Wnt/β-catenin and TGF-β/Smad2/3 signaling pathway, which were cluster analyzed using IPA and DAVID. Our results showed that PCDHGA9 could induce apoptosis, cell cycle arrest and autophagy to suppress GC cell proliferation and growth. Additionally, PCDHGA9 was found to inhibit the EMT process and decrease migration and invasion in GC cells. More importantly, our results provide mechanistic insight, showing that PCDHGA9 might antagonize the canonical Wnt pathway and inhibit GC cell proliferation by interacting or combining with β-catenin and decreasing its nuclear translocation. Moreover, PCDHGA9 could reduce the EMT process and suppress tumor metastasis through TGF-β/smad2/3 signaling in GC cells. Thus, the present study illuminates the importance and underlying mechanisms of PCDHGA9 in GC proliferation and metastasis.

### PCDHGA9 may represent a novel prognostic biomarker in GC

For confirmation, we demonstrated that the expression of PCDHGA9 was much lower in GC tissues compared with the corresponding normal mucosae. IHC staining results revealed significant correlations between low PCDHGA9 expression and GC pathological characteristics, including TNM stage, UICC stage, distant metastasis, histological differentiation, and relapse, among others. Kaplan–Meier analysis further revealed that PCDHGA9 might play an important role in GC pathogenesis and prognosis. Thus, our findings suggest PCDHGA9 as a novel biomarker of a highly malignant GC phenotype with poor survival.

### PCDHGA9 plays a significant role in cadherin interaction and carcinogenesis

Previous studies of Pcdhs have focused on their functions in neuronal differentiation, neuronal migration, axon outgrowth, dendrite arborization, synapse formation and stabilization, reflecting the precise and coordinated control of cell–cell interaction through Pcdhs^7–10^. However, few studies have examined the roles of Pcdhs in carcinogenesis. Recently, the aberrant expression of PCDHGA9 has been reported in some cancers with unfavorable prognosis, such as acute lymphoblastic leukemia, nasopharyngeal carcinoma, and astrocytoma^11–13^. However, more thorough analyses remain scarce, and the impact and mechanism of Pcdhs in cancer remain unclear. PCDHGA9 is one of the members of the Pcdh-γ gene clusters, and until recently, studies of PCDHGA9 had not been reported.

### PCDHGA9 reduces the EMT process and suppresses tumor metastasis through TGF-β/Smad2/3 signaling in GC cells

In vitro assays suggested that the overexpression or knockdown of PCDHGA9 could suppress or promote GC cell wound healing, migration and invasion activity. Furthermore, compared with PCDHGA9 overexpression, the control group showed increased peritoneal nodules and organic metastases in peritoneal xenografts models, consistent with the results in vitro. These data indicated that PCDHGA9 inhibited GC cell migration and invasion. Furthermore, the results of western blot and confocal immunofluorescence analyses suggested that the overexpression or knockdown of PCDHGA9 expression generated changes in EMT related proteins. Thus, we inferred that PCDHGA9 was associated with the EMT process in GC cells.

A number of signaling pathways trigger EMT, such as the TGF-β/Smad^14^, Notch^15^, RTK^16^, and Wnt signaling pathways^17^. Thus, we analyzed our microarray data to acquire information concerning EMT through IPA. This analysis indicated that the TGF-β/Smad2/3 pathway was strongly associated with EMT activation, and the proteins in the TGF-β/Smad2/3 pathway were co-regulated with PCDHGA9. Therefore, we next explored the roles of PCDHGA9 in TGF-β/Smad2/3 signaling during EMT. TGF-β signaling is activated when the cytokine interacts with transmembrane receptors of type I (TGFβ RI) or type II (TGFβ RII)^18^. Interaction with TGFβ RI leads to the phosphorylation of Smad2/3, which translocates to the nucleus and regulates the expression of target genes, such as Snal1, Slug, and ZEB1^19^. We demonstrated that the overexpression of PCDHGA9 could reduce the phosphorylation of Smad2/3 and suppress the nuclear translocation of pSmad2/3 to regulate the transcription of Snail1. Moreover, TGF-β1 strongly increased the migration and invasion of GC cells, whereas PCDHGA9 overexpression could restrain this process (Supplementary Figure 4). These results suggest that PCDHGA9 modulates EMT through TGF-β/Smad2/3 signaling.

### PCDHGA9 inhibits GC cell proliferation by interacting with β-catenin, decreasing its nuclear translocation, and antagonizing the canonical Wnt pathway

We demonstrated that PCDHGA9 inhibits GC cell proliferation and growth in vitro and in vivo. Enhanced expression of PCDHGA9 generated smaller tumor volumes in subcutaneous xenografts. Programmed cell death is mediated through an intracellular program, and apoptosis and autophagy are both forms of programmed cell death that are executed in distinct patterns^20^. Previous studies have proposed interconnections between apoptosis and autophagy to suppress cancer cell growth, whereby autophagy serves as a back-up system when apoptosis is disabled^21–24^. Indeed, several members of Pcdhs are known to inhibit cancer cell proliferation and growth^12^. We found that apoptosis and autophagy were induced through PCDHGA9 overexpression. Moreover, our results revealed that the upregulation of PCDHGA9 resulted in G_1_ cell cycle arrest and decreased the expression of cyclin D1 and c-myc. Additionally, inducing cell cycle arrest is a major strategy employed in traditional cancer therapies^25^.

The DNA array analysis via IPA showed that PCDHGA9 expression was considerably decreased, and some crucial molecules of the Wnt/β-catenin pathway, including β-catenin, LEF, TCF, MMP7, cyclin D1 (refs.^26, 27^) and others, were simultaneously altered. Moreover, DAVID analysis implied that the *p*-value of the Wnt signaling pathway was minimal, highlighting it as the most recommended cluster pathway^28^. The Wnt/β-catenin signaling pathway plays significant roles in tumorigenesis through regulating the transcription of downstream genes to impact cell cycle, apoptosis, proliferation, migration, and invasion^29^. Thus, we explored the effects of PCDHGA9 on canonical Wnt signaling. However, the confocal results showed that PCDHGA9 overexpression led to the redistribution of β-catenin. Interestingly, compared with the control group, β-catenin located on the membrane was increased, whereas β-catenin transferred to nucleus was decreased. This unusual phenomenon was puzzling. Why was β-catenin located on the membrane when PCDHGA9 was overexpressed? When Wnt pathway activates, dissociative β-catenin becomes stable and translocates to nucleus but has no association with the cell membrane^30^.

The cell adhesion complex and the Wnt pathway share the employment of β-catenin^31^. This intersection evokes an intriguing debate as to whether or not the Wnt pathway and cadherins compete through the utilization of β-catenin^31, 32^. Indeed, simple model organisms and experimental operations in cells have revealed that β-catenin signaling and cadherin expression are interrelated^33, 34^. For example, the upregulated expression of cadherin can antagonize β-catenin signaling activity in numerous systems^35, 36^. In contrast, reduced cadherin expression in developing tissues intensifies β-catenin signaling^37^. These discoveries evidently show how cadherins can, in general, function as stoichiometric inhibitors of the Wnt/β-catenin pathway. Pcdhs constitute the largest group of cadherins, although their impacts on nuclear signaling remain unclear. Chen reported that Pcdh–PC combines with β-catenin and enhances cell sensitivity to apoptosis^38^. However, further study is required to uncover the relationships among these anti-tumorigenic mechanisms in the context of PCDHGA9.

We hypothesized that PCDHGA9 could inhibit GC cell proliferation and growth via bonding to β-catenin and antagonizing canonical Wnt signaling in GC. Indeed, we observed that the enhanced expression of PCDHGA9 could suppress nuclear β-catenin accumulation, but no altered total β-catenin expression was observed. Moreover, PCDHGA9 was negatively correlated with cyclin D1, MMP7, and c-myc, which are the downstream target genes of Wnt/β-catenin signaling. In addition, the disruption of β-catenin using XAV-939 inhibited GC cell proliferation and colony formation in control cells significantly more than in PCDHGA9-overexpressing cells, suggesting that the Wnt/β-catenin pathway plays an important role during GC tumorigenesis in the context of PCDHGA9.

### Conclusion

The present study showed that PCDHGA9 was downregulated in human GC cell lines and tumor tissues and that PCDHGA9 overexpression could inhibit GC cell proliferation, migration, and invasion both in vivo and in vitro. Moreover, negative PCDHGA9 expression could serve as an independent prognosis biomarker. Furthermore, we found that PCDHGA9 suppresses EMT through TGF-β/Smad2/3 signaling and inhibits proliferation via interacting with β-catenin to antagonize the canonical Wnt pathway, revealing its role as a novel therapeutic target in GC prevention and treatment.

Notably, the present study only examined subcellular localization through western blotting and confocal microscopy, and we did not directly determine whether PCDHGA9 and β-catenin constitute a complex or not. Importantly, there might be some interconnections between Wnt/β-catenin and EMT signaling, as shown in Supplementary Figure 5 and analyzed via IPA, which we did not clearly elaborate in the present study. However, further complete experimental schemes adopting additional technologies, such as co-immunoprecipitation or Glutathione S-transferase (GST) pull-down assays, are needed to uncover this information.

## Materials and methods

### Microarray assay

Total RNA was isolated from cell culture using TRIzol reagent (Invitrogen, California, USA) according to the manufacturer’s instructions. Sample labeling and array hybridization were performed using the GeneChip Hybridization Wash and Stain Kit (Affymetrix, California, USA). The GeneChip PrimeView Human Gene Expression Array cartridge enables expression profiling using probe sets with an emphasis on established, well-annotated content. The sequences used in the design of the array were selected from RefSeq version 36, UniGene database 219, and full-length human mRNAs from GenBank™. The PrimeView Human Gene Expression Array comprises >530,000 probes covering >36,000 transcripts and variants, which represent >20,000 genes mapped through RefSeq or via UniGene annotation. The expressed sequence tag (EST) and mRNA sequences used in the present study were clustered and assembled to generate consensus sequences that represent alternative splice forms. Each assembly was subsequently analyzed for orientation and alternative 3′ end evidence. After quantile normalization of the raw data, the mRNAs that were detected in at least three out of six samples were chosen for further data analysis. Differentially expressed mRNAs with statistical significance between the two groups were identified through *p*-value/false discovery rate (FDR) filtering.

### Patient and tissue samples

Patient-derived samples were obtained in accordance with the guidelines approved through the institutional review boards of Shanghai General Hospital Affiliated to Shanghai JiaoTong University. Procedures were executed according to the approved guidelines. A total of 83 GC patient specimens were obtained at the time of surgery between July 2006 and April 2007. Written informed consent was obtained from all subjects, and the study was conducted according to the World Medical Association Declaration of Helsinki. No patients received chemotherapy, radiotherapy, or other related antitumor therapies before surgery. At least two certified pathologists confirmed all diagnoses, and the tumor stage and grade classification was based on pathological findings according to the International Union Against Cancer guidelines. OS and DFS rates were defined as the interval from the initial surgery to clinically or radiologically confirmed recurrence/metastasis and death, respectively. The clinicopathological characteristics of the patients are summarized in Table 1.

### Analysis of biological networks and validated target genes in the cDNA array

All enrichment analyses of gene-Gene ontology (GO) term and bio-pathways were statistically identified using DAVID Bioinformatics Resources 6.8 (ref. ^28^) and IPA^39^. The data were obtained from the aforementioned cDNA arrays, and the gene IDs and fold-changes were imported into DAVID or IPA software. IPA is based on the Ingenuity Knowledge Base and algorithmically generates bioinformatics, including top analysis-ready molecules, diseases and functions, toxin functions, canonical pathways and regulator effect networks, within the up-loaded data set. Subsequently, each molecule, pathway, or network receives a *p*-value or *p*-value range, suggesting its importance.

### Quantitative real-time polymerase chain reaction (qRT-PCR)

Total RNA was isolated from cell cultures or primary tissues and adjacent normal mucosa of GC patients using TRIzol reagent (Invitrogen) according to the manufacturer’s instructions. The HiScript Q RT SuperMix for qPCR (+gDNA wiper) kit (Vazyme, Nanjing, China) was used for the reverse transcription of 2 μg of RNA according to the manufacturer’s instructions. qRT-PCR was performed using the ChamQ SYBR qPCR Master Mix (Vazyme) kit. The following primers were used for qRT-PCR: PCDHGA9, forward: 5′-GCTCATTTCGGTGGAAGAT-3′ and reverse 5′ CACTGGGCTAAACAGAGAT 3′; GAPDH, Forward 5′ GGGAAGGTGAAGGTCGGAGT 3′ and Reverse 5′-GGGGTCATTGATGGCAACA-3′. Relative quantities (Δ cycle threshold (Ct values)) were obtained by normalizing to glyceraldehyde-3-phosphate dehydrogenase (GAPDH). Each PCR product was run in triplicate, and the relative PCDHGA9 mRNA levels were calculated using the 2^-∆∆Ct^ method.

### Western blot analysis

Tissue and total cell lysates were extracted using RIPA lysis buffer with the protease inhibitor phenylmethanesulfonyl fluoride (Beyotime Biotechnology, Jiangsu, China), and cytomembrane or nuclear proteins were extracted using the Membrane and Cytosol Protein Extraction Kit and the Nuclear and Cytoplasmic Protein Extraction Kit, respectively (Yeasen, Shanghai, China). The protein concentration was measured using the BCA protein assay kit (Beyotime Biotechnology, Jiangsu, China) according to the manufacturer’s instructions. Equivalent amounts of protein (30 μg) were separated on 10% sodium dodecyl sulfate-polyacrylamide gel electrophoresis (SDS-PAGE) gels and subsequently transferred onto polyvinylidene difluoride (PVDF) membranes (Millipore, Billerica, USA) using standard protocols. LC3B was separated using a 15% SDS-PAGE gel, followed by electro-transfer onto a PVDF membrane. The membranes were sequentially blocked in 5% skim milk in tris-buffered saline and tween 20 (TBST) buffer for 1.5 h at room temperature, followed by incubation with primary antibodies at 4 °C overnight. After incubation with a secondary antibody for 1 h at room temperature, the proteins were detected using ECL regent (Millipore, Billerica, USA). Primary antibodies specific to anti-GAPDH (1:1000, Santa Cruz, CA, USA), anti-MMP7 (1:500, Santa Cruz), anti-MMP9 (1:500, Santa Cruz), c-myc (1:000, Cell Signaling Technology, Massachusetts, USA), cyclin D1 (1:1000, Cell Signaling Technology), E-cadherin (1:800, Cell Signaling Technology), Vimentin (1:800, Cell Signaling Technology), PCDHGA9 (1:500, Abcam, London, UK), β-catenin (1:1000, Abcam), N-cadherin (1:1000, Abcam), Smad2 (1:1000, Abcam), Smad3 (1:1000, Abcam), pSmad2 (1:1000, Abcam), pSmad3 (1:1000, Abcam), Caspase-9 (1:000, Cell Signaling Technology), cleaved-caspase-9 (1:000, Cell Signaling Technology), caspase-3 (1:000, Cell Signaling Technology), cleaved-caspase-3 (1:000, Cell Signaling Technology), LC3B (1:000, Cell Signaling Technology), ATG5 (1:000, Cell Signaling Technology), Beclin-1 (1:000, Cell Signaling Technology), SQSTM1/p62 (1:300, Abcam).

### TMA construction and IHC staining

A total of 83 cases retrieved from Shanghai General Hospital were included in the TMA, including primary GC tumors tissue and corresponding distant normal mucosa. Hematoxylin and eosin staining was used to validate tissue morphology and cores (2.0 mm diameter) were punched from the paraffin blocks. To guarantee equal reaction conditions, paired cores obtained from the same patient were placed next to each other. For the overall consideration of the results, we graded the IHC results according to the staining intensity and region as previously described^40^. Specifically, the percentage of positive cells was divided into five grades (percentage scores): <10% (0), 10–25% (1), 25–50% (2), 50–75 (3), and >75% (4). The intensity of staining was divided into four grades (intensity scores): no staining (0), light brown (1), brown (2), and dark brown (3). PCDHGA9 staining positive was determined using the following formula: overall score = percentage score × intensity score. An overall score of 0–3 indicated negative expression; 4–6 indicated weak expression; and 8–12 indicated strong expression. Two pathologists assessed all specimens to avoid bias. The corresponding primary antibodies were: PCDHGA9 (1:250, Abcam), Ki-67 (1:500, Cell Signaling Technology).

### Cell culture and transfection

The human GC cell lines AGS, HGC-27, MKN-28, MKN-45, MGC-803, BGC-823, and SGC-7901 and normal human gastric mucosa cell line GES-1 were obtained from the Type Culture Collection of the Chinese Academy of Sciences (Shanghai, China). The cell lines mentioned above were cultured in 1640 medium supplemented with 10% fetal bovine serum (FBS) (Gibco, California, USA).

### RNA interference

We used a lentivirus-based vector for the RNA interference experiments. Oligonucleotides targeting PCDHGA9 (PCDHGA9 short hairpin RNAs (shRNAs), 5′-GCGGAAGATTAGTCCTGCTAT-3′ and 5′-CCTGCAAGTGACTGACATCAA-3′) were selected and cloned into the Plvx vector. Recombinant lentiviral plasmids were co-transfected into 293T cells with the packaging plasmids pMD2G, pRSV-REV, and pMDLg-pRRE. After 72 h, the viral supernatants were passed through 0.45-μm filters and subsequently used to infect MGC-803 and AGS cells in the presence of 6 μg/ml Polybrene (Sigma-Aldrich, Darmstadt, Germany).

### Plasmid constructs and transfection

A PCDHGA9 expression plasmid (pCMV-PCDHGA9) was constructed with synthetic oligonucleotides and the pCMV vector. SGC-7901 cells were infected with pCMV-PCDHGA9 or a negative-control (NC) vector using Lipofectamine 2000 (Invitrogen).

### Cell proliferation and plate colony formation assays

Cell proliferation was detected with the Cell Counting Kit-8 assay (Yeasen) according to the manufacturer’s instructions. At 24, 48, 72, and 96 h, cell viability was evaluated by measuring the absorbance at 490 nm on the Gen5 micro-plate reader (BioTek, Ermont, USA). Plate colony formation assays were performed as previously described^29^. All assays were independently performed in triplicate.

### Cell apoptosis assays

Apoptosis assays were performed using SGC-7901-vector and SGC-7901-PCDHGA9 expression cells using the Annexin V-PE apoptosis detection kit (eBioscience, Vienna, Austria) according to the manufacturer’s instructions.

### Cell cycle analysis

The Cell Cycle and Apoptosis Analysis kit (Beyotime, Jiangsu, China) was used to evaluate the influence of overexpression of PCDHGA9 on the cell cycle of GC cells. SGC-7901-PCDHGA9 or control group cells were seeded onto 6-well plates. After culture for 24 h, these cells were harvested and fixed with alcohol at 4 °C for 12 h. Subsequently, cell cycle analysis performed using a flow cytometer (BD Biosciences, New Jersey, USA).

### Scratch wound-healing assays

SGC-7901-PCDHGA9, MGC-803-KD, and control group cells were grown to full confluence on six-well plates. After 12 h, a uniform scratch was generated down the center of the well using a micropipette tip, followed by washing once with phosphate-buffered saline (PBS). Images were captured at 0-, 18-, 36-, 54-, and 72-h intervals, and wound widths were quantified and compared with baseline values.

### Cell migration and invasion assays

The in vitro migration and invasion assay was performed using transwell 24-well Boyden chambers (Corning, New York, USA) with an 8.0-μm pore size polycarbonate membrane without (migration) or with (invasion) Matrigel. Each group of cells (2 × 10^4^/chamber) was plated in the upper chamber in 200 μl serum-free media for 36 h, whereas the bottom chamber contained 600 μl media supplemented with 10% FBS as a chemoattractant. Cells that migrated and invaded to the reverse side of the chamber inserts were fixed using methyl alcohol and stained with 0.1% crystal violet. Experiments were repeated in triplicate.

### Indirect immunofluorescence microscopy

Cells were seeded onto sterile coverslips in plates at 60–70% confluence. The next day, the cells were washed once with PBS, fixed in 4% paraformaldehyde for 20 min and permeabilized with 0.1% Triton X-100 for 6 min. After blocking for 1 h with blocking buffer (1% bovine serum albumin in PBS buffer, pH 7.4), the cells were incubated with antibodies against to Smad2, pSmad2, E-cadherin, N-cadherin, LC3B, or SQSTM1/p62 overnight, followed by incubation with a fluorophore-conjugated secondary antibody (Abcam) for an additional 2 h. Cell nuclei were stained with 4′,6 diamidino-2-phenylindole (DAPI) for 1 min. A confocal laser-scanning microscope (TCS SP8; Leica, Wetzlar, Germany) was used to collect fluorescence images. Primary antibodies specific to anti-E-cadherin (1:200, Cell Signaling Technology), N-cadherin (1:400, Abcam), Smad2 (1:250, Abcam), and pSmad2 (1:1000, Abcam).

### In vivo tumor xenograft and metastasis experiments

The animal assays were approved through the Institutional Animal Care and Use Committee of Shanghai General Hospital, and 4-week-old male BALB/c nude mice were used for the animal studies. For the subcutaneous tumor growth assay, 200 μl of 2 × 10^6^ SGC-7901-PCDHGA9, SGC-7901-KD, or control cells was subcutaneously injected into the groin of nude mice, respectively. There were six or seven mice in each group. Tumor diameters were measured every 3 days. Tumor volume was calculated using the formula (volume = length × width^2^ × 1/2). Four weeks later, all mice were sacrificed, and the tumors were harvested. After fixing with 4% paraformaldehyde, the tumors were embedded in paraffin for IHC staining.

For peritoneal metastatic assays, 1 × 10^6^ GC cells, including either PCDHGA9-overexpressing or knockdown or control cells, were injected into the abdominal cavity of nude mice. Each group included three mice. After 4 weeks, all mice were euthanized, and the peritoneal metastatic nodules in the peritoneal cavity of the mice were observed.

### Chemicals

XAV-939 and Z-VAD-FMK were obtained from MedChemExpress (New Jersey, USA), TGF-β1 was purchased from Peprotech (USA).

### Statistical analysis

Data were analyzed using the SPSS 22.0 statistical software package (SPSS, Chicago, IL, USA). The Student’s *t*-test was applied to estimate the differences in PCDHGA9 mRNA level between GC tissues and corresponding normal mucosa. The statistical significance between clinicopathological variables and PCDHGA9 expression was determined using the *χ*^2^ test or Fisher’s exact test. The Kaplan–Meier method with the log-rank test employed for the comparison of differences was used to calculate survival curves. The hazard ratio (HR) with 95% confidence interval in the Cox proportional hazards regression was utilized to measure the hazard risk of individual factors for OS and DFS. For all tests, a* p*-value < 0.05 was considered statistically significant.

## Electronic supplementary material

supplementary Figure 1

supplementary Figure 2

supplementary Figure 3

supplementary Figure 4

supplementary Figure 5

Supplementary Figure Legends
