# Supplementary figures and images for: PCDHGA9 acts as a tumor suppressor to induce tumor cell apoptosis and autophagy and inhibit the EMT process in human gastric cancer
Source: Cell Death Dis. 2018 Jan 18;9(2):27. doi: 10.1038/s41419-017-0189-y (PMC5833845; doi:10.1038/s41419-017-0189-y)

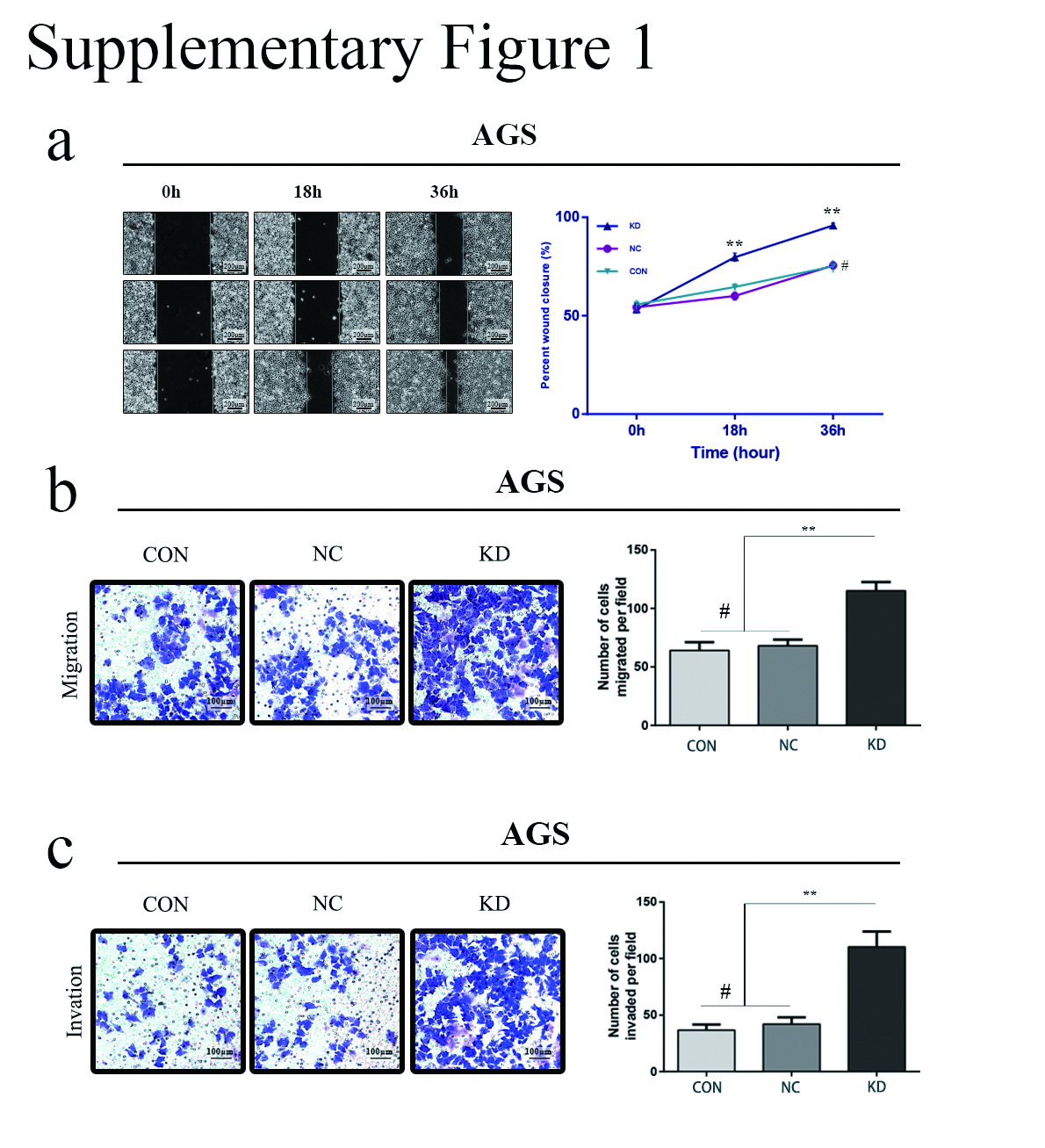

Supplement: Supplementary file 1 — supplementary Figure 1 [file 41419_2017_189_MOESM1_ESM.tif]

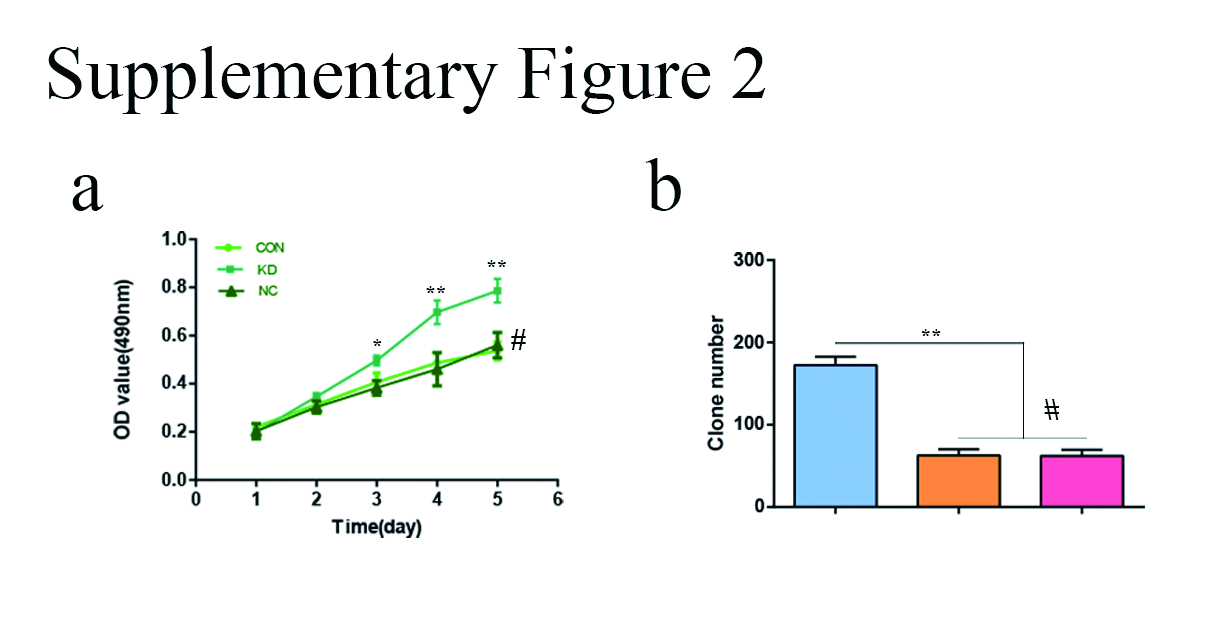

Supplement: Supplementary file 2 — supplementary Figure 2 [file 41419_2017_189_MOESM2_ESM.tif]

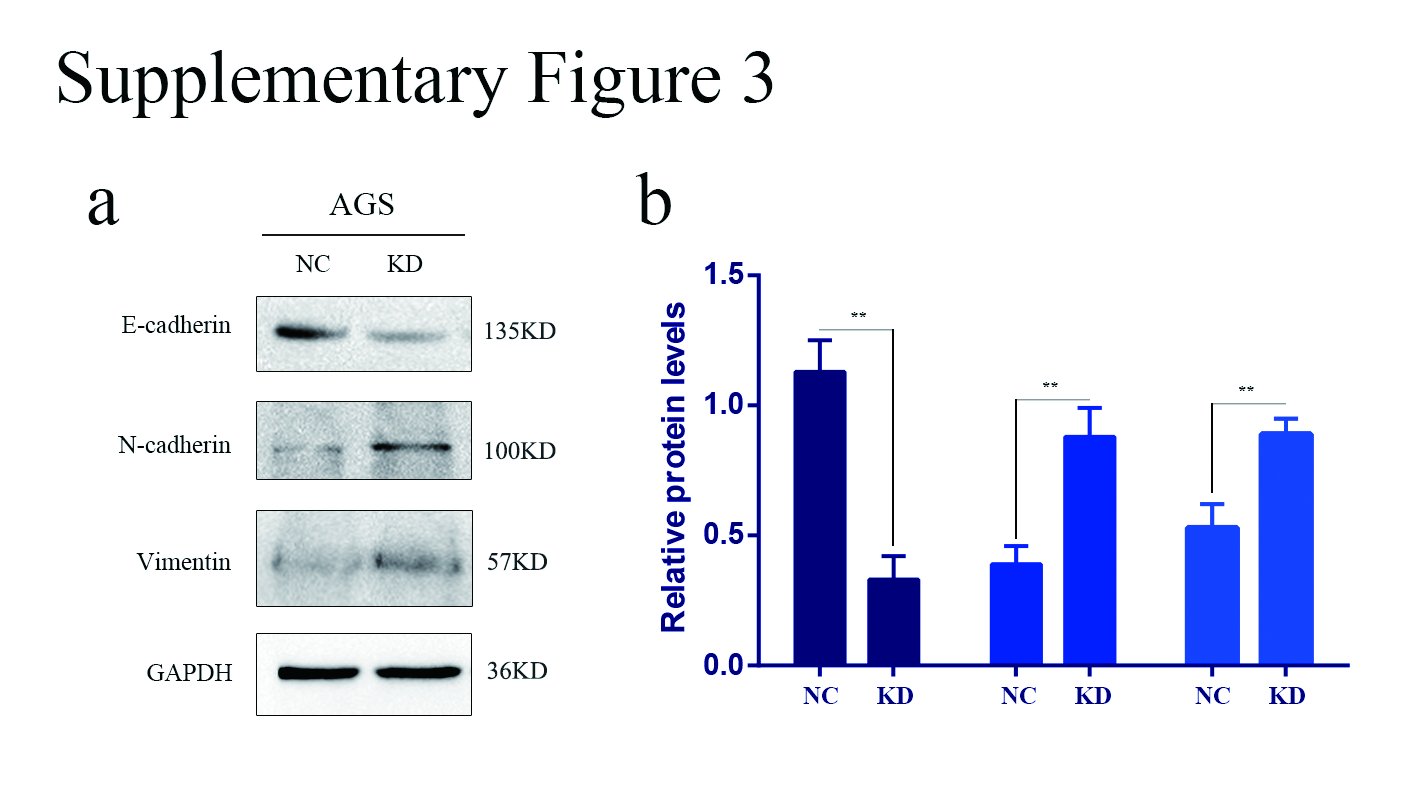

Supplement: Supplementary file 3 — supplementary Figure 3 [file 41419_2017_189_MOESM3_ESM.tif]

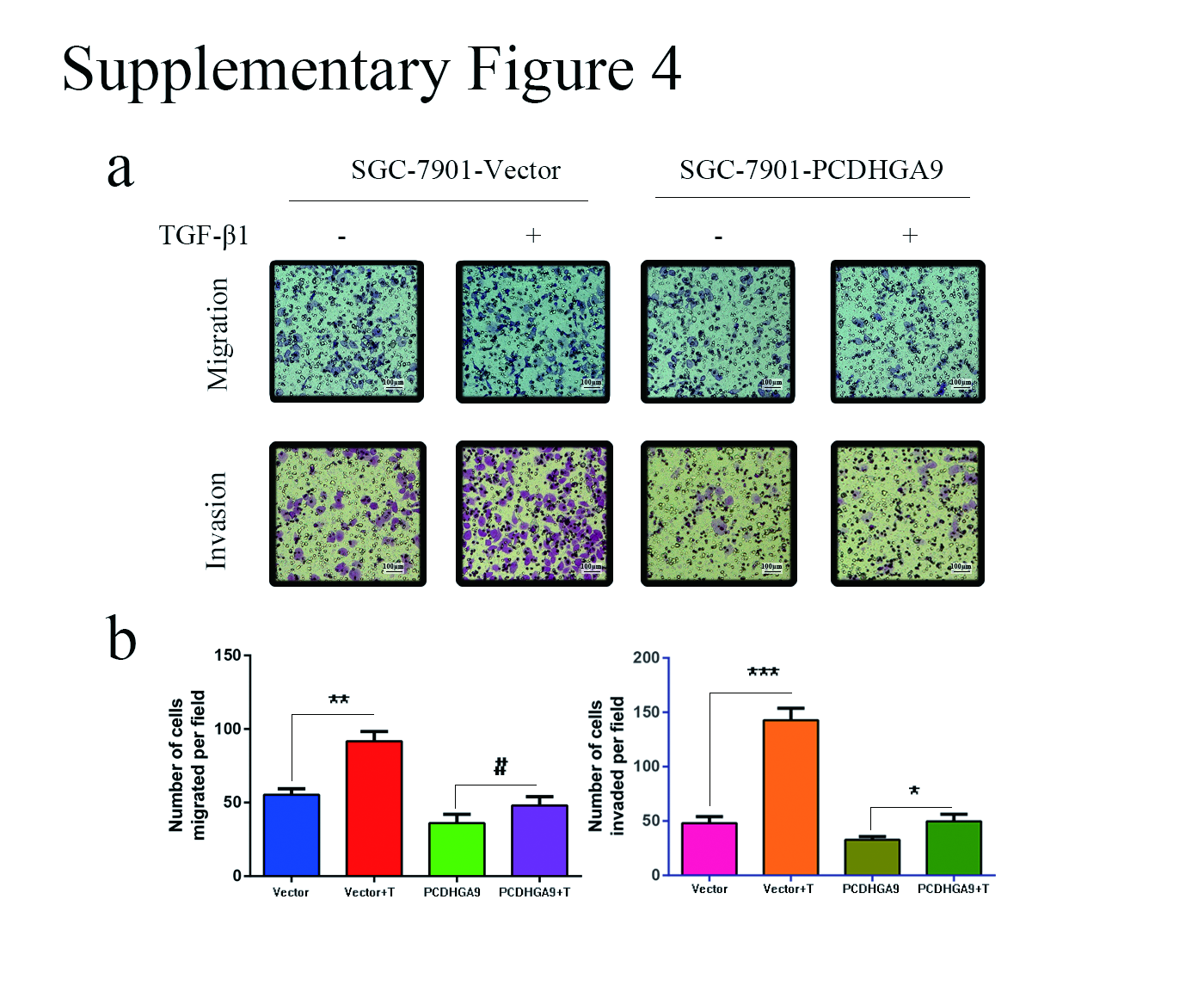

Supplement: Supplementary file 4 — supplementary Figure 4 [file 41419_2017_189_MOESM4_ESM.tif]

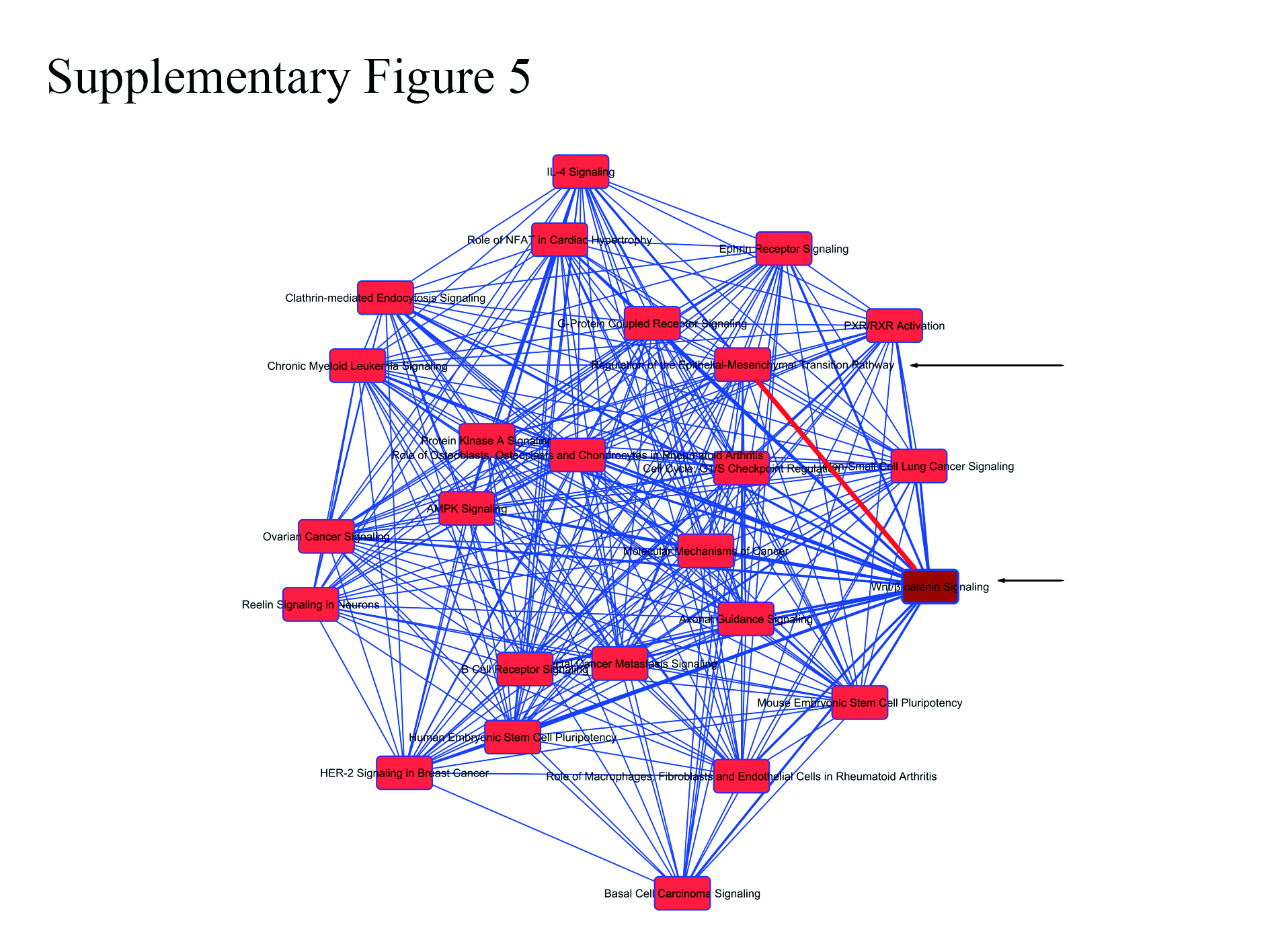

Supplement: Supplementary file 5 — supplementary Figure 5 [file 41419_2017_189_MOESM5_ESM.tif]
