# Supplementary material for: PCDHGA9 acts as a tumor suppressor to induce tumor cell apoptosis and autophagy and inhibit the EMT process in human gastric cancer
Source: Cell Death Dis. 2018 Jan 18;9(2):27. doi: 10.1038/s41419-017-0189-y (PMC5833845; doi:10.1038/s41419-017-0189-y)
Supplement: Supplementary file 6 — Supplementary Figure Legends [file 41419_2017_189_MOESM6_ESM.docx]

**Supplementary Figure Legends**

**Supplementary Figure 1:** The migration and invasion assays were performed in AGS PCDHGA9-overexpressing and control cells. (** p＜0.01, # p＞0.05)

**Supplementary Figure 2:** knock down of PCDHGA9 elevated AGS proliferation: (a) CCK8 assays, (b) Colony formation assay. (** p < 0.01).

**Supplementary Figure 3:** Western blot analysis of EMT markers (E-cadherin, N-cadherin and Vimentin) in the PCDHGA9 knockdown AGS cells compared with the control group. GAPDH was used to normalize protein expression.

**Supplementary Figure 4:** The migration and invasion assays were performed in SGC-7901 PCDHGA9-overexpressing and control cells before and after adding TGF-β1. (*** p＜0.001, ** p＜0.01, * p＜0.05, # p＞0.05)

**Supplementary Figure 5:** The signaling enrichment analysis and overlapping canonical pathways from cDNA array analysis via IPA showed an association between Wnt/β-catenin and EMT regulation pathway.
